# ARF: Connecting senescence and innate immunity for clearance

**DOI:** 10.18632/aging.100813

**Published:** 2015-09-25

**Authors:** Alper Y. Kearney, Benedict Anchang, Sylvia Plevritis, Dean W. Felsher

**Affiliations:** ^1^ Division of Oncology, Departments of Medicine and Pathology, Molecular Imaging Program, Stanford University, Stanford, CA 94305, USA; ^2^ Department of Radiology, Stanford University, Stanford, CA 94305, USA; ^3^ Current Address: Genitourinary Medical Oncology, The University of Texas, MD Anderson Cancer Center, Houston, TX 77030, USA

**Keywords:** Myc inactivation, p19arf, innate immunity, senescence

## Oncogene addiction and MYC

Cancers depend on one or more ubiquitously activated oncogenes to survive and maintain their tumorigenic phenotype. The MYC oncogene is critically important in a variety of hematological malignancies, including Burkitt's lymphoma, acute lymphoblastic leukemia, and multiple myeloma. Cancers' reliance on few oncogenes, aptly termed oncogene addiction, presents an exploitable vulnerability. Indeed, targeted therapies inhibiting key oncogenes in a multitude of cancer types are routinely used in clinic [1]. However, in many cases cancers either do not respond to oncogene inhibition therapies or eventually become resistant following an initial response phase. Resistance to oncogene inhibition therapies and ensuing tumor recurrence are major contributors to cancer-specific mortality.

Experimental model systems of MYC oncogene inactivation in various cancers have revealed molecular mechanisms of oncogene addiction, escape from oncogene dependence resulting in tumor recurrence [2]. Inactivation of the MYC oncogene in mouse models of cancer induces proliferative arrest, apoptosis and/or cellular senescence, as well as the shutdown of angiogenesis; thereby, enabling tumor clearance and sustained tumor regression [3, 4]. However, in some cases, tumors reoccur [5].

Recently, we gained insight into the mechanism of tumor recurrence. We found that when either of two tumor suppressor genes, ARF or p53, is lost or mutated, tumor recurrence rapidly always occurs [6]. We previously reported that p53 loss results in tumor recurrence by maintaining tumor vasculature, despite MYC inactivation, through upregulation of angiogenesis inhibitor TSP1 [3]. Further, we have described that INK4A/ARF loss or RB1 loss results in tumor recurrence through abrogation of cellular senescence. We found distinct mechanisms through which loss of ARF or p53 leads to tumor recurrence in a mouse model of T-ALL [6]. We identified that ARF has a p53-independent effect on the ability of MYC inactivation to elicit expression of genes involved innate immune activation associated with a marked decrease in the recruitment of macrophages.

## p53-dependent and p53-independent effects of ARF

**Figure 1:**
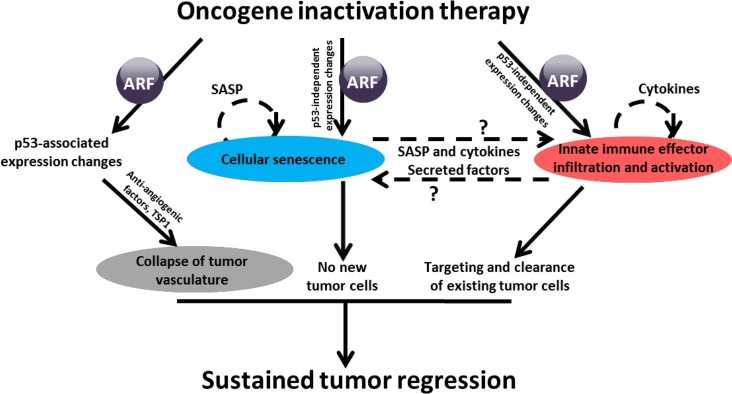
ARF loss interferes with expression of genes associated with senescence and innate immune system MYC inactivation, in the presence of ARF, triggers gene expression changes in pathways associated with senescence as well as macrophage and innate immune cell activation and infiltration. Microarray analysis revealed that ARF loss precludes these changes, which we propose may be how ARF loss blocks macrophage infiltration. SASP: Senescence-associated secretory phenotype.

We found that ARF loss prevented MYC inactivation from inducing cellular senescence through a p53 independent mechanism [6]. Our observations are consistent with a prior study that ARF regulates cellular senescence in p53-independent manner in melanoma [7]. Further, we found that ARF loss, but not p53 loss, blocked macrophage infiltration into regressing tumors. In contrast, loss of p53 had modest effect on cellular senescence and no effect on macrophage infiltration, but instead allowed maintenance of tumor vasculature, even in regressing tumors. Thus, the loss of either ARF or p53 enables tumor recurrence, albeit through different mechanisms. Therefore, ARF's effects on angiogenesis are likely to be p53-dependent, while ARF's effects on senescence and innate immune infiltration and activation seem to be p53-independent (Figure 1).

The mechanism through which ARF regulates innate immunity is not clear. Analysis of gene expression revealed a signature of genes involved in innate immunity [6]. ARF, itself, is not thought to be directly a transcription factor, but is known to interact with or regulate transcription factors, such as MYC and p53 [8-10]. We considered ARF may be diminishing MYC's transactivation or transrepression capabilities; however, analysis of MYC target genes did not support this [6].

## ARF and immunotherapy

Emerging immunotherapies have intensified interest in the therapeutic role of the innate and adaptive immune system. Biologics targeted at tumor cells for enabling antibody-dependent cell-mediated cytoxicity (ADCC) rely on innate immune system for elimination of cancer cells [11]. Since ARF loss in tumors influences infiltration and activation of innate immune effectors, ARF status may be important to immunotherapies. In particular, this may be relevant for drugs that utilize ADCC, such as anti-ERBB2 antibody trastuzumab and anti-CD20 antibody rituximab.

Cells undergoing senescence secrete factors that bolster the senescent phenotype in both autocrine and paracrine fashion by engaging innate immune system [12]. ARF loss is associated with expression changes in senescence and innate immune system-related genes as well as defects in macrophage infiltration into tumors. Our results are consistent with a previous report that ARF deficiency interferes with macrophage activation, cytokine production, and inflammation [13]. Conceivably, ARF null tumors may present a different secretome profile, which may abrogate SASP-dependent autocrine and paracrine positive feedback loops and elicit incomplete targeting and clearance of tumor cells by immune effectors. We have yet to identify an individual factor that may mediate effects of ARF on innate immune system; however, likely candidates include TGFbeta, IL-1alpha/beta, and NFkB among others. TGFbeta and pro-inflammatory cytokine IL-1 have been previously implicated in the reinforcement and spread of senescence [14, 15]. Transactivation activity of NFkB, common regulator of SASP and innate immunity, is intriguingly altered by ARF [16, 17]; however, whether ARF loss alters SASP and cytokine and chemokine profiles in T-ALL is not known.

Our research has uncovered ARF as a common regulator of senescence and tumor clearance that appears to require the innate immune system. However, there are several questions awaiting answers. First, we have identified innate immune-related gene expression changes due to ARF loss; yet direct causality of these changes in macrophage infiltration into regressing tumors has not been established. Second, the mechanism by which cellular senescence is dependent upon macrophage recruitment is unclear. Third, whether ARF loss in tumors affects other immune infiltrates, such as NK cells, monocytic and granulocytic myeloid cells, or even adaptive immune cells, needs to be investigated. Fourth whether ARF's effect on innate immune-related genes and recruitment of macrophages extends to other types of cancers remains to be determined. Finally, it is of paramount interest whether ARF status may influence efficacy of immunotherapies.

## References

[1] Sharma SV, Settleman J (2007). Oncogene addiction: setting the stage for molecularly targeted cancer therapy. Genes & development.

[2] Felsher DW, Bishop JM (1999). Reversible tumorigenesis by MYC in hematopoietic lineages. Molecular cell.

[3] Giuriato S, Ryeom S, Fan AC, Bachireddy P, Lynch RC, Rioth MJ, van Riggelen J, Kopelman AM, Passegue E, Tang F, Folkman J, Felsher DW (2006). Sustained regression of tumors upon MYC inactivation requires p53 or thrombospondin-1 to reverse the angiogenic switch. Proceedings of the National Academy of Sciences of the United States of America.

[4] Wu CH, van Riggelen J, Yetil A, Fan AC, Bachireddy P, Felsher DW (2007). Cellular senescence is an important mechanism of tumor regression upon c-Myc inactivation. Proceedings of the National Academy of Sciences of the United States of America.

[5] Karlsson A, Giuriato S, Tang F, Fung-Weier J, Levan G, Felsher DW (2003). Genomically complex lymphomas undergo sustained tumor regression upon MYC inactivation unless they acquire novel chromosomal translocations. Blood.

[6] Yetil A, Anchang B, Gouw AM, Adam SJ, Zabuawala T, Parameswaran R, van Riggelen J, Plevritis S, Felsher DW (2015). p19ARF is a critical mediator of both cellular senescence and an innate immune response associated with MYC inactivation in mouse model of acute leukemia. Oncotarget.

[7] Ha L, Ichikawa T, Anver M, Dickins R, Lowe S, Sharpless NE, Krimpenfort P, Depinho RA, Bennett DC, Sviderskaya EV, Merlino G (2007). ARF functions as a melanoma tumor suppressor by inducing p53-independent senescence. Proceedings of the National Academy of Sciences of the United States of America.

[8] Cleveland JL, Sherr CJ (2004). Antagonism of Myc functions by Arf. Cancer cell.

[9] Gregory MA, Qi Y, Hann SR (2005). The ARF tumor suppressor: keeping Myc on a leash. Cell cycle.

[10] Weber JD, Taylor LJ, Roussel MF, Sherr CJ, Bar-Sagi D (1999). Nucleolar Arf sequesters Mdm2 and activates p53. Nature cell biology.

[11] Scott AM, Wolchok JD, Old LJ (2012). Antibody therapy of cancer. Nature reviews Cancer.

[12] Krizhanovsky V, Xue W, Zender L, Yon M, Hernando E, Lowe SW (2008). Implications of cellular senescence in tissue damage response, tumor suppression, and stem cell biology. Cold Spring Harbor symposia on quantitative biology.

[13] Traves PG, Lopez-Fontal R, Luque A, Hortelano S (2011). The tumor suppressor ARF regulates innate immune responses in mice. Journal of immunology.

[14] Tasdemir N, Lowe SW (2013). Senescent cells spread the word: non-cell autonomous propagation of cellular senescence. The EMBO journal.

[15] van Riggelen J, Muller J, Otto T, Beuger V, Yetil A, Choi PS, Kosan C, Moroy T, Felsher DW, Eilers M (2010). The interaction between Myc and Miz1 is required to antagonize TGFbeta-dependent autocrine signaling during lymphoma formation and maintenance. Genes & development.

[16] Chien Y, Scuoppo C, Wang X, Fang X, Balgley B, Bolden JE, Premsrirut P, Luo W, Chicas A, Lee CS, Kogan SC, Lowe SW (2011). Control of the senescence-associated secretory phenotype by NF-kappaB promotes senescence and enhances chemosensitivity. Genes & development.

[17] Rocha S, Garrett MD, Campbell KJ, Schumm K, Perkins ND (2005). Regulation of NF-kappaB and p53 through activation of ATR and Chk1 by the ARF tumour suppressor. The EMBO journal.

